# A combination of Nottingham prognostic index and IHC4 score predicts pathological complete response of neoadjuvant chemotherapy in estrogen receptor positive breast cancer

**DOI:** 10.18632/oncotarget.13549

**Published:** 2016-11-24

**Authors:** Weige Tan, Wei Luo, Weijuan Jia, Gehao Liang, Xinhua Xie, Wenbo Zheng, Erwei Song, Fengxi Su, Chang Gong

**Affiliations:** ^1^ Guangdong Provincial Key Laboratory of Malignant Tumor Epigenetics and Gene Regulation and Breast Tumor Center, Sun Yat-Sen Memorial Hospital, Sun Yat-Sen University, Guangzhou, China; ^2^ Department of Breast Surgery, The First Affiliated Hospital of Guangzhou Medical University, Guangzhou, China; ^3^ Sun Yat-sen University Cancer Center, State Key Laboratory of Oncology in South China, Collaborative Innovation Center for Cancer Medicine, Guangzhou, China; ^4^ Collaborative Innovation Center for Cancer Medicine, Sun Yat-Sen University, Guangzhou, China

**Keywords:** breast cancer, pathologic complete response, neoadjuvant chemotherapy, Nottingham prognostic index, IHC4

## Abstract

Pathologic complete response (pCR) prediction after neoadjuvant chemotherapy (NAC) is important for clinical decision-making in breast cancer. This study investigated the predictive value of Nottingham prognostic index (NPI), Immunohistochemical four (IHC4) score and a new predictive index combined with them in estrogen-positive (ER+) breast cancer following NAC. We retrospectively gathered clinical data of 739 ER+ breast cancer patients who received NAC from two cancer centers. We developed a new predictive biomarker named NPI+IHC4 to predict pCR in ER+ breast cancer in a training set (n=443) and validated it in an external validation set (n=296). The results showed that a lower IHC4 score, NPI and NPI+IHC4 were significantly associated a high pCR rate in the entire cohort. In the study set, NPI+IHC4 showed a better sensitivity and specificity for pCR prediction (AUC 0.699, 95% CI 0.626-0.772) than IHC4 score (AUC 0.613, 95% CI 0.533-0.692), NPI (AUC 0.576, 95% CI 0.494-0.659), tumor size (AUC 0.556, 95% CI 0.481-0.631) and TNM stage (AUC 0.521, 95% CI 0.442-0.601). In the validation set, NPI+IHC4 had a better predictive value for pCR (AUC 0.665, 95% CI 0.579-0.751) than IHC4 score or NPI alone. In addition, ER+ patients with lower IHC4, NPI and NPI+IHC4 scores had significantly better DFS in both study and validation sets. In summary, NPI+IHC4 can predict pCR following NAC and prognosis in ER+ breast cancer, which is cost-effect and potentially more useful in guiding decision-making regarding NAC in clinical practice. Further validation is needed in prospective clinical trials with larger cohorts of patients.

## INTRODUCTION

Neoadjuvant chemotherapy (NAC) has been a standard therapeutic approach for patients with locally advanced operable, primarily non-operable or inflammatory breast cancers [1]. Although several trials comparing different NAC regimens have failed to demonstrate an association between pathologic complete response (pCR) rates and improved outcome [2], many studies have shown that achieving pCR after NAC predicts a long survival, independent of treatment regimen [3–7]. However, only 5 to 38% of breast tumors attain a pCR [8] and patients without pCR may run the risk of cancer progression during NAC therapy [9, 10], suggesting that predicting pCR is important in clinical practice.

Estrogen receptor-positive (ER+) breast cancer accounts for approximately 60% of all primary breast cancer cases [11]. Albeit a lower risk of tumor recurrence compared to other molecular subtypes, around 20% of ER+ patients may develop local/distant recurrences after treatment, and younger patients (age≤ 40 years) are found to have a much higher 5-year breast cancer specific-mortality rate (BCSM), mounting up to 40%, than elder patients [12]. More importantly, compared to ER negative and triple-negative subgroups, the ER+ subgroup benefits less from cytotoxic chemotherapies [13, 14] and achieve a lower pCR rate after NAC [15, 16]. Several specific biomarkers have been identified and used to predict a pCR for ER+ breast cancer. On one hand, a couple of traditional clinicopathological variables including tumor size, nuclear grade and Ki-67 may be provide predictive information regarding NAC [9, 15, 17]. On the other hand, multi-gene models have been highly correlated with the achievement of pCR in ER+ patients [18–20]. However, both of these factors are subject to issues related to inadequate predictive performance, partly due to lopsided and incomplete information provided by incorporated variables, and are limited for feasible clinical application. Based on this, our attention has been focused on developing surrogate biomarkers that incorporate macro-anatomic features and molecular information for improving the predictive performance for pCR and identification of ER+ individuals who may receive the most benefit from NAC.

The Nottingham prognostic index (NPI) is a clinicopathological classification system based on tumor size, histological grade, and lymph-node status widely used in Europe and the United Kingdom for breast cancer prognostication [21–24]. Despite its utility, it is also acknowledged that the widely used NPI has limited literature to determine its effect on the pCR of breast cancer in the context of NAC. A combined four immunohistochemical marker score (IHC4 score), which includes the estrogen receptor (ER), progesterone receptor (PR), human epidermal growth factor receptor 2 (HER2) and Ki-67, has been clinically validated to evaluate the risk of early distant recurrence in ER+ breast cancer patients [16, 25, 26]. IHC4 scoring is attractive because it has been demonstrated to be as informative as the 21-gene recurrence score (RS) and is substantially less expensive than the RS [20, 25, 27] in ER+ patients. The overall impression is that few papers in the literature have explored the value of the IHC4 for pCR. Only one study from a small cohort showed that a lower IHC4 score was associated with an increased probability of pCR in ER+ breast cancer patients [28]. Thus, it can be seen that the combination of NPI and IHC4 score covers the traditional anatomical pathological factors and gene information of patients and provides more comprehensive information of individuals.

The primary aim of this study was to assess and compare the predictive value of the NPI, IHC4 score and a combination of these systems at the time of diagnosis for a pCR to NAC. As a secondary aim, we investigated the association between NPI, IHC4 or the combination of systems and disease-free survival (DFS) in ER+ patients who received NAC. In the present study, we demonstrated that the combination of NPI and IHC4 score (NPI+IHC4) prior to NAC outperformed NPI or IHC4 score alone and each single clinicopathological factor in predicting pCR (ypT0/is ypN0) and further validated it in an external, independent group. The patients with a lower IHC4 score and NPI+IHC4 prior to NAC had better DFS in the ER-positive patients.

## RESULTS

### Patient characteristics within the study set

The baseline clinicopathological features and treatments of the study set are shown in Table 1 (Study set). In the study set, there were 87 (19.6%) patients who developed local recurrence or distant metastasis during the follow-up. The median age of the patients was 47.3 years. With a median follow-up of 68.45 months (ranging from 9.87 to 162.5 months), the 5-year and 10-year DFS were 81.5% and 76.9%, respectively. Clinically, 361 (81.5%) patients had tumor size ≤5cm and 346 (78.1%) patients had positive axillary lymph nodes prior to NAC. Most tumors were PR-positive (247 of 443, 55.8%), HER2-negative (275 of 443, 62.1%) and Ki-67>14% (299 of 443, 67.5%).

**Table 1:** Associations between the clinicopathological variables and pCR

Characteristics		Study set (n=443)			Validation set (n=296)		
		Non-pCR	pCR	P-value	Non-pCR	pCR	P-value
Age	≤ 40y	98(86.0%)	16(14.0%)	0.746	44(84.6%)	8(15.4%)	0.824
	>40y	288(87.5%)	41(12.5%)		210(86.1%)	34(13.9%)	
Tumor size	> 5cm	77(93.9%)	5(6.1%)	0.044	41(85.4%)	7(14.6%)	0.774
	≤ 5cm	309(85.6%)	52(14.4%)		213(85.9%)	35(14.1%)	
LN status	Negative	84(86.6%)	13(13.4%)	0.864	109(84.5%)	20(15.5%)	0.863
	Positive	302(87.3%)	44(12.7%)		145(86.8%)	22(13.2%)	
TNM stage	I	7(87.5%)	1(12.5%)	0.316	46(85.2%)	8(14.8%)	0.505
	II	148(85.1%)	26(14.9%)		131(85.1%)	23(14.9%)	
	III	231(88.5%)	30(11.5%)		77(87.5%)	11(12.5%)	
PR	Negative	164(83.7%)	32(16.3%)	0.063	38(86.4%)	6(13.6%)	0.948
	Positive	222(89.9%)	25(10.1%)		216(85.7%)	36(14.3%)	
HER2	Negative	247(89.8%)	28(10.2%)	0.04	195(85.5%)	33(14.5%)	0.418
	Positive	139(82.7%)	29(17.3%)		59(86.8%)	9(3.9%)	
ki-67	≤14%	128(88.9%)	16(11.1%)	0.545	126(87.5%)	18(12.5%)	0.468
	>14%	258(86.2%)	41(13.8%)		128(84.2%)	24(15.8%)	
Grade	1	56(86.2%)	9(13.8%)	0.231	20(87.0%)	3(13.0%)	0.256
	2	168(90.3%)	18(9.7%)		107(89.9%)	12(10.1%)	
	3	162(84.4%)	30(15.6%)		127(82.5%)	27(17.5%)	
NAC	E-based	93(87.7%)	13(12.3%)	0.52	86(92.5%)	9(9.5%)	0.160
	ET-based	293(86.9%)	44(13.1%)		168(83.6%)	33(16.4%)	
Surgery	BCS	116(81.70%)	26(18.30%)	0.023	97(75.8%)	31(24.2%)	0.012
	MRM	270(89.70%)	31(10.30%)		156(93.4%)	11(6.6%)	
NPI	< 3.4	75(79.8%)	19(20.2%)	0.043	57(78.1%)	16(21.9%)	0.022
	3.4∼5.4	197(88.7%)	25(11.3%)		123(85.4%)	21(14.6%)	
	>5.4	114(89.8%)	13(10.2%)		74(93.7%)	5(6.3%)	
IHC4 score	Q1	88(79.3%)	23(20.7%)	0.012	84(80.0%)	21(20.0%)	0.047
	Q2	196(88.7%)	25(11.3%)		97(86.6%)	15(13.4%)	
	Q3	102(91.9%)	9(8.1%)		73(92.4%)	6(7.6%)	

Abbreviations: pCR, pathological complete response; LN, lymph node; ER, estrogen receptor; PR, progesterone receptor; HER2, human epidermal growth factor receptor 2; NAC, neoadjuvant chemotherapy; E-based, anthracycline-based regiment; ET, anthracycline and taxanes-based regiment; BCS, breast conserving surgery; MRM, mamomodified radical mastectomy.

With regard to the treatments administered, 106 patients (23.9%) received an anthracycline-based regiment without taxanes (EC or CEF); 337 patients (76.1%) received anthracyclines-axanes based regiments (CET or EC followed by T). Only 11 of the 168 Her2-positive patients (6.5%) were treated with preoperative chemotherapy combined with trastuzumab. Among the remaining HER2-positive patients (n=157) who did not receive neoadjuvant trastuzumab treatment, only 5 patients (0.03%) received adjuvant trastuzumab treatment following surgery. Following the completion of NAC, 142 patients (32.1%) received breast conserving surgery; 301 patients (67.9%) received a modified radical mastectomy (MRM), and a pathological assessment was performed on the final surgical specimens.

We calculated the IHC4 score and the Nottingham prognosis Index (NPI) of the primary tumor for each patient. More than half (222 in 443, 50.1%) of the patients had NPI 3.4∼5.4, while 94 (21.2%) patients had an NPI of less than 3.4, and 127 (28.7%) patients had an NPI score greater than 5.4.

### The associations between the study clinicopathological variables and pCR

A total of 57 patients (12.9%) achieved pCR (ypT0/is ypN0). Baseline characteristics according to pCR status was shown in Table 1 (Study set). In univariate analysis, baseline characteristics in both pCR and no pCR subgroups were similar with respect to age, LN status, clinical TNM stage, PR and Ki67 status, grade, regimen of chemotherapy, use of Herceptin. Patients who achieved a pCR had a lower IHC4 score (*P* =0.012), lower NPI (*P*=0.043) and smaller tumor size (≤ 5cm vs >5cm; *P*=0.044). Her2-positive patients seemed to get pCR more easily (positive vs negative, *P*=0.04). Surgical procedures was associated with pCR in univariate analysis (*P*=0.023). In multivariable analysis, independent predictors of pCR included tumor size (≤5cm vs. >5cm: odds ratio [OR], 3.4; 95% confidence interval [CI], 1.11-10.44; *P* =0.03), TNM stage (Stage II∼III vs. I: OR, 3.24; 95% CI 1.30 - 8.05; *P* =0.01), IHC4 score (Q1 vs.Q3:OR, 5.49; 95% CI, 1.04 - 28.87; *P*=0.04; Q2 vs.Q3: OR, 3.20; 95% CI, 0.89 - 11.56; P=0.08) and NPI (<3.4 vs. >5.4:OR, 18.82; 95% CI, 4.19 - 84.63; *P*<0.001; 3.4∼5.4 vs. >5.4: OR, 3.32; 95% CI, 1.23 - 8.92; *P*=0.02) (Table 2).

**Table 2:** Multivariable model and adjusted odds ratio of variables considered for pCR of ER+ breast cancer patients

Variables	Odds ratio	95% CI	P value
Age			
≤40y vs. >40y	1.44	0.72 - 2.85	0.30
Grade			
Grade III vs.I	3.00	0.94 - 9.58	0.06
Grade II vs.I	2.09	0.78 - 5.60	0.14
TNM stage			
Stage II∼III vs. I	3.24	1.30 - 8.05	0.01
NPI			
<3.4 vs. >5.4	18.82	4.19 - 84.63	<0.001
3.4∼5.4 vs. >5.4	3.32	1.23 - 8.92	0.02
Tumor size			
≤5cm vs. >5cm	3.40	1.11 - 10.44	0.03
LN status			
Negative vs. Positive	1.28	0.61 - 2.66	0.52
PR			
Negative vs. Positive	1.08	0.52 - 2.25	0.84
Her2			
Negative vs. Positive	1.03	0.40 - 2.65	0.95
Ki67			
*>14%* vs. ≤14%	1.28	0.66 - 2.48	0.46
IHC4 score			
Q1 vs.Q3	5.49	1.04 - 28.87	0.04
Q2 vs.Q3	3.20	0.89 - 11.56	0.08

Abbreviations: NPI, Nottingham Prognosis Index; LN, lymph node; PR, progesterone receptor; HER2, human epidermal growth factor receptor 2; IHC4, A combined four immunohistochemical marker score.

### Generation of a new NPI+IHC4 scoring system for predicting pCR

Since multivariable analysis showed four variables including tumor size, TNM, NPI and IHC4 score were independent predictors of pCR, we further undertook ROC analysis to test and compared the predictive value of these four independent predictors. The results indicated that IHC4 had better predictive value (AUC 0.613, 95% CI 0.533-0.692) than tumor size (AUC 0.556, 95% CI 0.481-0.631), TNM stage (AUC 0.521, 95% CI 0.442-0.601) and NPI (AUC 0.576, 95% CI 0.494-0.659). These four factors retained in the model were non- overlapping and relatively independent and ORs for them was all of similar magnitude. Binary indicators were assigned to presenting macro-anatomic features and molecular information retained in the model. Therefore, we combined IHC4 score with NPI by variable assignment and generated a new scoring system defined as NPI+IHC4. In this new scoring system, patients were scored 1∼3 point if their IHC4 scores were Q1∼Q3 respectively. Patients scored 1∼3 point if their NPI were <3.4, 3.4∼5.4 or >5.4 respectively. The overall NPI+IHC4 score was then determined by summing the points as listed in Table 3, which ranged between 2∼6 point.

**Table 3:** Point Assignments for the IHC4+NPI Scoring System

Variable	Score
IHC4	
Q1	1
Q2	2
Q3	3
NPI	
>5.4	1
3.4∼5.4	2
< 3.4	3

**Figure 1:**
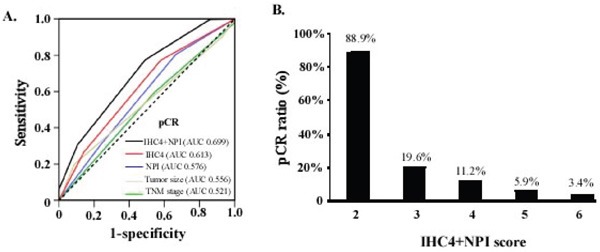
**A**. Comparison of predictive value between NPI+IHC4 scoring system and other predictors in the Study set. AUC=area under curve. ROC=receiver operator characteristic. **B**. pCR Ratio of different subgroups stratified by NPI+IHC4.

**Figure 2:**
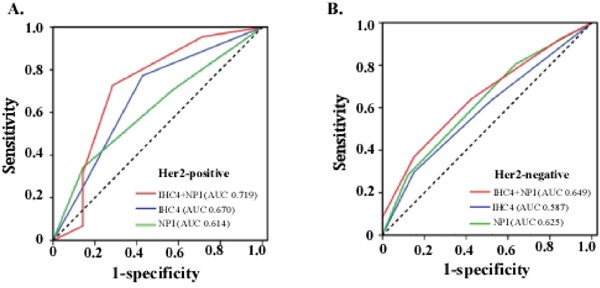
Predictive value of NPI+IHC4 scoring system, IHC4 score and NPI in subgroups stratified by Her2 **A**. Her2-positive; **B**. Her2-negative.

NPI+IHC4 score showed a better sensitivity and specificity for pCR prediction (AUC 0.699, 95% CI 0.626-0.772), which enhanced the predicting ability of either IHC4 score or NPI significantly (Figure 1A). The patients with lower NPI+IHC4 score were much more likely to achieve pCR (*P*<0.001) (Figure 1B). Further stratified analysis showed that NPI+IHC4 score had predictive value for pCR in both Her2-negative (AUC 0.719) and Her2-positive (AUC 0.649) subgroups, which was better than the IHC4 or NPI alone Figure 2.

### Survival analysis of study set

**Figure 3:**
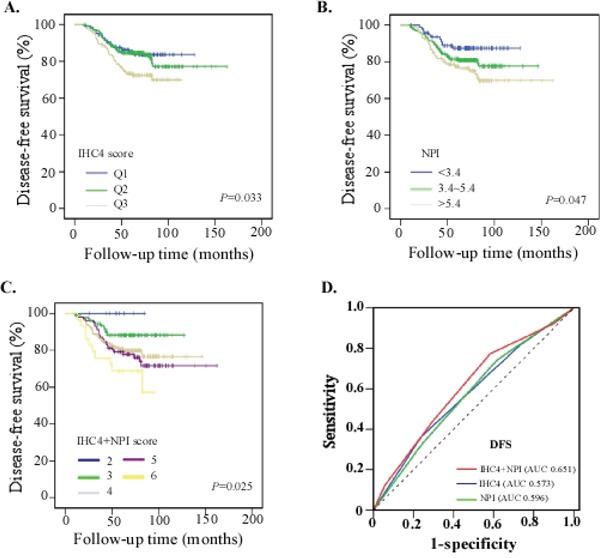
Survival analysis of predictors for the Study set **A**. Kaplan-Meier survival curves for patients with different IHC4 score; **B**. Kaplan-Meier survival curves for patients with NPI; **C**. Kaplan-Meier survival curves for patients with different NPI+IHC4 score; **D**. Comparison of the prognostic accuracy of NPI+IHC4 with IHC4 score and NPI alone in the Study set. We calculated *p* values using the log-rank test.

Compared with the non-pCR patients, the patients with a pCR had a longer disease-free survival (DFS) (*P*=0.048) (Supplementary Figure S2). In this study set, both IHC4 score, NPI score and NPI+IHC4 score were associated with DFS. The patients with lower IHC4 score (*P*=0.033), NPI (*P*=0.047) and NPI+IHC4 (*P*=0.025) score had significantly better DFS (Figure 3A-3C). ROC analysis showed that the prognostic value for DFS of NPI+IHC4 (AUC 0.651) was better than IHC4 score (AUC 0.573) or NPI alone (AUC 0.596) (Figure 3D).

### Validation of predictors of pCR and prognosis

A total of 296 primary ER-positive breast cancer patients from Sun Yat-Sen Cancer center (SYSUCC) were enrolled into validation study retrospectively. The same enrolled criteria was applied to the validation set and its clinicopathological characteristics were matched with a study set from Sun Yat-Sen Memorial hospital (Table 1, Validation set). There were 66 (22.3%) patients who developed local recurrence or distant metastasis in the study set during the follow-up. The median age of the patients was 48.4 years. With a median follow-up of 68.45 months (range from 8.1 to 120.4 months), the 5-year and 10-year DFS were 78.4% and 74.3%, respectively.

**Figure 4:**
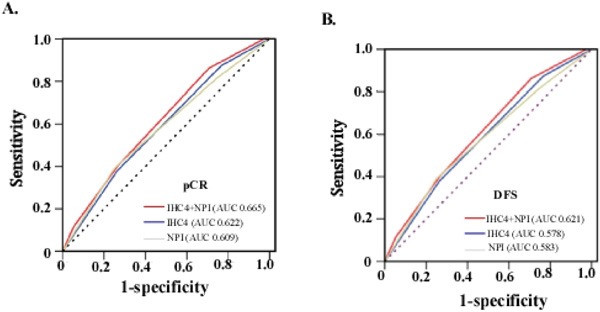
Comparison of predictive value and prognosis accuracy of NPI+IHC4, IHC4 and NPI in validation set **A**. Predictive value for pCR; **B**. Prognostic accuracy for DFS.

In this validation set, IHC4 score and NPI were verified and found to associate with pCR and prognosis significantly. Patients with lower IHC4 score (pCR rate, Q1, 20%; Q2, 13.4%; Q3, 7.6%; *P*=0.047), NPI (pCR rate, NPI <3.4, 21.9%; NPI 3.4∼5.4, 14.6%; NPI >5.4, 6.3%; *P*=0.022) and NPI+IHC4 score (pCR rate of 2∼6 score, 29.6%, 20.2%, 12.5%, 6.6%, 4.8%; *P*=0.011) were more likely to achieve pCR (Table 1, Validation set) and better prognosis (Supplementary Figure S3). Moreover, compared to IHC4 score or NPI alone, ROC analysis verified that NPI+IHC4 score had a better predictive value for pCR (AUC 0.665, 95%CI 0.579-0.751) (Figure 4A) and disease-free survival (AUC 0. 621) (Figure 4B).

## DISCUSSION

In the present study, we have developed and validated a novel predictive index based on the combination of NPI and IHC4 scores to improve the prediction of pCR following NAC in 739 patients with ER+ breast cancer. In the entire cohort, a lower IHC4 score, NPI and NPI+IHC4 were significantly associated a high pCR rate. The NPI+IHC4 exhibited an increased predictive accuracy for pCR compared with NPI or IHC4 score alone and the other clinicopathological factors. In addition, significant differences in DFS could be established for patients when stratified by the NPI+IHC4 and either of them. To our knowledge, this is the first study to demonstrate the predictive role of the combination of NPI and IHC4 score in predicting pCR of BC patients in the context of NAC.

pCR prediction is of high important because a valid prediction of residual tumor absence may strongly influence clinical decision-making. Various clinical parameters and molecular biomarkers, such as age, Body Mass Index (BMI), tumor stage, histological type, hormone receptor, HER2 status and Ki-67 expression level, have been correlated with a pCR [3, 4, 16]. However, when a single biomarker is applied, none of these biomarkers predicts a pCR with sufficient accuracy due to limited information. Based on our findings, NPI+IHC4, which integrates four molecular biomarkers and three clinicpathological features, increased the predictive value for a pCR and outperpformed any single factor. This predictor was subsequently validated in an external independent group. Our findings demonstrate NPI can complement the predictive value of IHC4 score by employing a variable assignment method. The reasons why the performance of combination outperform each of them alone are as follow: Firstly, NPI showed lower performance than IHC4 score (AUC 0.614 *vs*. 0.670), suggesting that the predictive value of anatomical pathological factors maybe less powerful than that of gene profiling. Secondly, since IHC4 score is as informative as 21-gene recurrence score in ER+ patients [16, 25, 26], and the prognostic and predictive information of the first generation signatures such as 21-gene stems almost exclusively from the degree of expression of proliferation-related genes [32], while incorporating NPI into IHC4 score provides added information, additional to proliferation. Thirdly, integration of anatomical pathological and molecular information facilitates more accurate assessment of the metastatic behavior, growth rate and genetic instability of breast cancers. The higher the score of NPI+IHC4 involved, the lower the pCR rate.

As described in the results, we analyzed the correlations of the IHC4 score with respect to the cancer subtype. Firstly, in this cohort, the patients with lower IHC4 scores or NPI+IHC4 had a higher pCR rate in ER-positive patients with a small size. Published studies have reported that patients with small tumors and low IHC4 score were more likely to obtain a pCR [4], which was consistent with our results. Secondly, according to clinical data, ER+ breast cancer is not as sensitive to chemotherapy as ER-negative patients [33, 34]. With respect to the regimens of NAC, both E-based and ET-based regimens showed similar effect on a pCR. These findings suggest that ER-positive tumors with lower IHC4 scores and small tumor size are more likely to respond to E-based regimens of NAC. Thirdly, anti- Her2 therapy is currently used widely in early her2-positive patients in adjuvant and neoadjuvant therapy, however, anti-Her2 therapy was not available for the majority of our study participants because of economic and insurance restrictions during that time in China. Of interest, stratified analysis showed that NPI+IHC4 had better predictive value for pCR than IHC4 score or NPI alone in both Her2-negative and Her2-positive subgroups, suggesting that this new combined score maybe play a potentially predictive role for pCR in her2-positive subgroup.

For ER+ breast cancer patients, there is a sharp decrease in disease-free survival during the first 3 to 5 years after diagnosis, and distant relapse after this time is much less common. In our present study, both IHC4 score, NPI and NPI+IHC4 were associated with DFS. Compared to IHC4 or NPI alone, ROC analysis verified that NPI+IHC4 had a better predictive value for DFS in both study and validation set. All these suggest that NPI+IHC4 might be a superior prognostic biomarker for ER+ breast cancer than NPI and IHC4 score. Further validation is needed in early patients who received adjuvant therapy.

Several limitations should be addressed. First, our study comprised the limitations inherent in a retrospective study. Further validation is needed in prospective clinical trials with larger cohorts of patients. Second, caution must be applied because of the small samples in several subgroup analyses. Third, few her2-positive patients received neoadjuvant or adjuvant trastuzumab therapy, which may have an impact on the pCR rate and survival [30]. This point may explain, in part, why the pCR rate was not as high in the present study as previously reported studies.

In summary, we developed and validated a new predictive biomarker named NPI+IHC4 in predicting pCR following NAC in ER+ breast cancer patients and demonstrated that NPI+IHC4 outperformed NPI or IHC4 score alone and other clinicopathological factors in predicting pCR.

## MATERIALS AND METHODS

### Patients

A total of 739 ER+ breast cancer patients were collected from the Breast Tumor Center, Sun Yat-sen Memorial Hospital of Sun Yat-sen University (SYSU) (Study set, n=443), and Sun Yat-sen University Cancer Center (SYSUCC) (Validation set, n=296) between January 2000 and November 2010. No patients exhibited distant-metastasis at the initial diagnosis. Patients who received NAC for less than 4 cycles or who did not have available IHC staining data, complete follow-up information or surgery followed by NAC were excluded. The detailed patient selection process of the study set is shown in Supplementary Figure S1. The eligible criteria for enrollment of patients was listed in the Additional Information section. The study protocol was approved by the independent ethical committee/institutional review board of SYSU and SYSUCC, and written informed consent regarding the scientific research was obtained from each participant prior to surgery. Patient records were anonymized and de-identified prior to analysis.

### Clinical data

Tumor clinical and pathological characteristics and treatment data were documented by the hospital. All treatment decisions for patients were in accordance with the most recent NCCN Breast Cancer Treatment Guidelines and China guidelines for the treatment of breast cancer. Follow-up information regarding local recurrences, distant metastases, and death was provided every three months following the initial diagnosis. Data, including clincopathological data and follow-up, were available for all patients.

### Quantification of NPI and IHC4 score

The Notttingham Prognosis Index (NPI) was calculated as follows: NPI=tumor size (cm)*0.2 +grade+ lymph-node points (negative nodes= 1 point; positive nodes, 1 to 3 positive =2 points; positive nodes, ≥4 =3 points). NPI can define 3 subsets of patients with different survival probabilities from breast cancer; good (≤3.4), moderate (3.41 - 5.4), and poor (>5.4) prognosis groups [29].

According to a published study [16], the IHC4 score was calculated using the following algorithms: IHC4 score= 94.7 × {-0.100ER10- 0.079PR10 +0.586Her2 + 0.240 In 1+10×Ki-67)}. ER was quantified using the H-score. The variable ER10 was obtained by dividing the H-score by 30 to obtain a variable with a range of 0 to 10. The PR was quantified by the percentage of cells that were stained positive, and this value was divided by 10 to obtain a variable between 0 and 10 (PR10). HER-2 was considered positive if the score was 3+ by IHC or 2+ by IHC and confirmed by fluorescence in situ hybridization amplification. The Ki-67 scores were recorded as the percentage of positively stained malignant cells. We identified the patients into three group by quartering, including quartile 1 [Q1, at the 25^th^ percentile of the IHC4 score], quartile 2 [Q2, at the 25^th^ to 75^th^ percentile of the IHC4 score] and quartile 3 [Q3, at the 75^th^ percentile of the IHC4 score].

### pCR assessment and prognostic endpoints

Carcinoma *in situ* was allowed and no evidence of tumor cell in the axilla was defined as pCR (ypT0/is ypN0), which is widely used in clinical practice, because the presence of residual ductal carcinoma in situ (DCIS) following preoperative therapy does not influence local recurrence or overall survival [30]. The disease-free survival (DFS) was used for prognostic assessment. DFS was measured from the date of radical operation to the date of recurrence (including locoregional recurrence and distant metastasis) or the last follow-up visit. The locoregional or distant recurrences were evaluated via physical examination and radiological imaging.

### Statistical analysis

Chi-square test was used to evaluate the associations among a pCR and clinicopathological characteristics. Multivariate analysis of the clinicopathological factors for a pCR among the baseline parameters was performed via logistic regression analysis. The ROC curves were employed to test the sensitivity and specificity of variables in predicting pCR and DFS. NPI+IHC4 scoring system was obtained through combined IHC4 score and NPI using the variable assignment method [31]. We used a Kaplan-Meier method to display the survival curves and log-rank test to compare the difference between subgroups. *P* < 0.05 was considered significant in all statistical analyses. All statistical analyses were performed using SPSS software (SPSS version 21, SPSS Inc., Chicago, IL, USA).

## CONCLUSIONS

NPI+IHC4 can predict pCR following NAC and prognosis in ER+ breast cancer. This study provide evidence that incorporating macro-anatomic features and molecular information to improve pCR prediction following NAC. NPI+IHC4 is cost-effect and maybe more useful in guiding decision-making regarding NAC in clinical practice.

## SUPPLEMENTARY DATA FIGURES


