# Supplementary material for: A combination of Nottingham prognostic index and IHC4 score predicts pathological complete response of neoadjuvant chemotherapy in estrogen receptor positive breast cancer
Source: Oncotarget. 2016 Nov 24;7(52):87312–22. doi: 10.18632/oncotarget.13549 (PMC5349990; doi:10.18632/oncotarget.13549)
Supplement: Supplementary file 1 [file oncotarget-07-87312-s001.pdf]

# A combination of Nottingham prognostic index and IHC4 score predicts pathological complete response of neoadjuvant chemotherapy in estrogen receptor positive breast cancer

## SUPPLEMENTARY DATA

### Eligible criteria of enrolled patients

1. Patients >18 years old, without any other malignant tumors before initial diagnosis of breast cancer.
2. Non-metastatic invasive ductal carcinoma (IDC).
3. IHC determined ER-positive breast cancer. We defined ER as positive if ≥1% of tumor cells is immunoreactive.
4. All the patients received NAC at least 4 cycles.
5. All the patients received endocrine therapy after surgery.
6. Essential clinicopathological status and follow-up information were available.
7. Patients diagnosed before 2000 were excluded because of unavailable IHC data; patients diagnosed after 2011 were excluded to ensure adequate follow-up of the study population.
8. The pathological diagnosis of IHC markers (ER, PR, Her2, Ki67) were re-assessed independently by two breast pathologists.

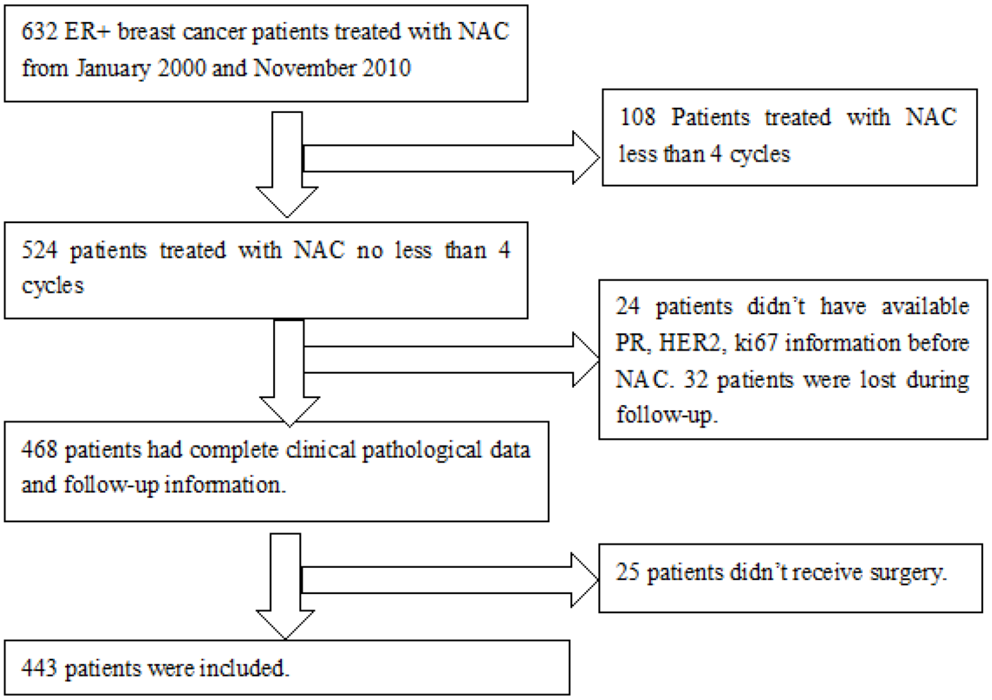


**Supplementary Figure S1: Flow chart of patient selection.**

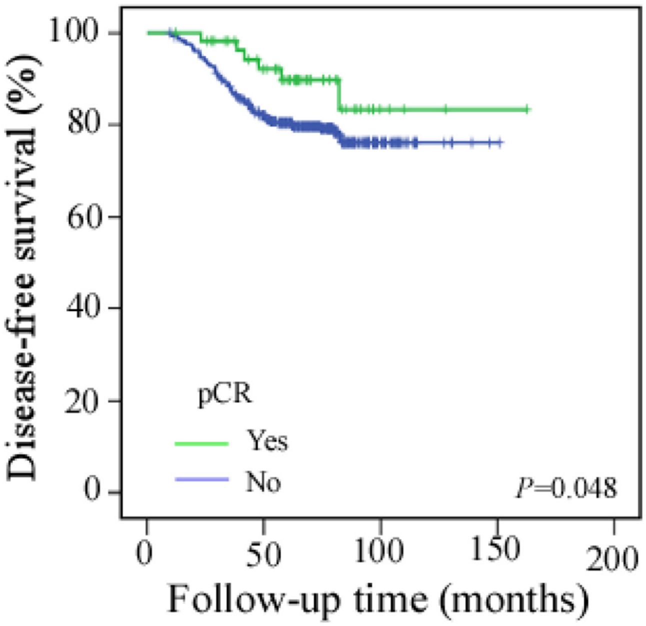


**Supplementary Figure S2: Kaplan-Meier survival analysis of pCR for study set.** We calculated the *p* value using the log-rank test.

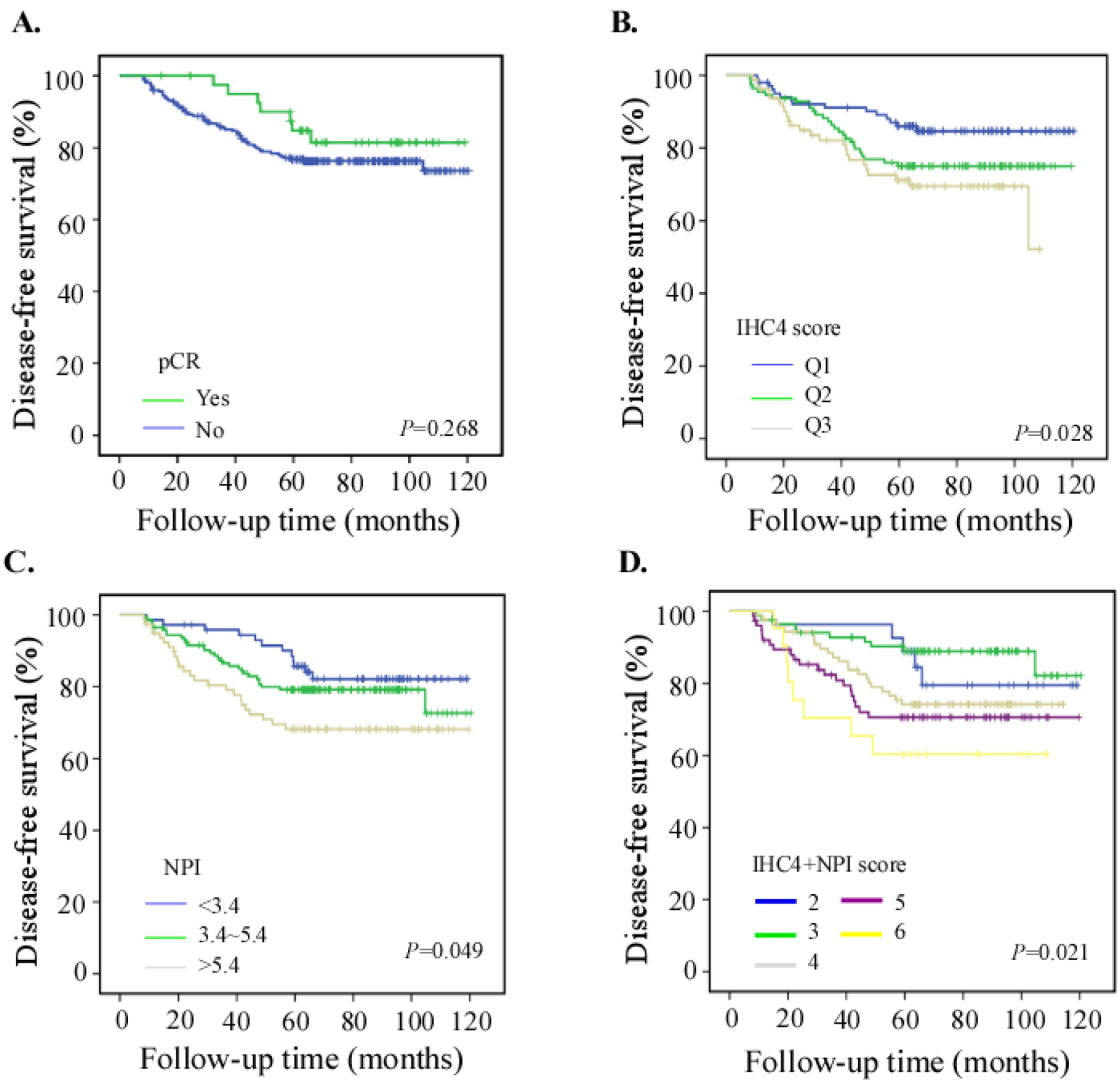


**Supplementary Figure S3: Kaplan-Meier survival analysis for validation set. A.** pCR. **B.** IHC4 score. **C.** NPI. **D.** IHC4+NPI score. We calculated *p* values using the log-rank test.
